# Renal Involvement in Thrombotic Thrombocytopenic Purpura: Is It Time to Challenge the Old Paradigm?

**DOI:** 10.7759/cureus.60259

**Published:** 2024-05-14

**Authors:** Lea Issa, Nicolas Sandakly, Georgio El Koubayati, Majd Khalil, Fady Haddad

**Affiliations:** 1 Internal Medicine, Faculty of Medicine, Lebanese University, Beirut, LBN; 2 Clinical Immunology, Faculty of Medicine, Lebanese University, Beirut, LBN; 3 Cardiology, Lebanese Hospital Geitaoui, Beirut, LBN; 4 Internal Medicine and Clinical Immunology, Lebanese Hospital Geitaoui, Beirut, LBN

**Keywords:** acute kidney injury, hemolysis, ttp pentad, thrombotic thrombocytopenic thrombocytopenia, acute kidney injury in ttp

## Abstract

Thrombotic thrombocytopenic purpura (TTP) is clinical-pathological entity characterized by microangiopathic hemolytic anemia associated with end-organ dysfunction. Traditionally, TTP was characterized by the classic pentad of fever, thrombocytopenia, hemolytic anemia, renal impairment, and neurological manifestations. However, this classic pentad is only observed in 40% of cases. We herein describe the case of a female patient who presented with epigastric pain and vomiting and found to have TTP without the classic pentad but with rapidly progressive renal dysfunction.

## Introduction

Thrombotic microangiopathy (TMA) represents a clinical-pathological entity characterized by microangiopathic hemolytic anemia accompanied by end-organ dysfunction. This condition encompasses two principal paradigms: hemolytic uremic syndrome (HUS) and thrombotic thrombocytopenic purpura (TTP) [1]. The first description of TTP dates back to 1925 when Moschcowitz reported a case of a 16-year-old girl who succumbed to fever, anemia, petechia, partial paresis of the left arm and leg, and facial paralysis within days of presentation. Autopsy findings revealed diffuse hyaline thrombosis of the terminal arterioles and capillaries [2]. Traditionally, TTP was characterized by the classic pentad of fever, thrombocytopenia, hemolytic anemia, renal impairment, and neurological manifestations. However, this classic pentad is only observed in 40% of cases [3]. Therefore, HUS and TTP are conventionally distinguished primarily by the extent of renal abnormality. Renal involvement has been well-described in typical HUS commonly described in children following infection with Shiga-toxin-producing Escherichia coli, as well as in atypical HUS, which occurs in childhood and adulthood, secondary to defect in complement regulation, leading to over-activation of complement alternative pathway and, subsequently, the activation of the coagulation cascade and thrombosis [4]. In contrast, little has been mentioned on kidney injury in TTP patients, even though frank renal failure has been reported, leading to a high rate of misdiagnosis and underdiagnosis. We herein describe the case of a female patient who presented with epigastric pain and vomiting and found to have TTP with rapidly progressive renal dysfunction.

## Case presentation

A 67-year-old female patient presented to the Lebanese Hospital Geitaoui University Medical Center (LHG-UMC) with one-day history of epigastric pain radiating to the shoulders, accompanied by nausea, vomiting, and anorexia. Prior to that day, the patient lived a normal active life. She denied any signs or symptoms of upper or lower respiratory tract infections or gastrointestinal or genitourinary infections. Her medical history is significant for hypothyroidism on levothyroxine 100 mcg once daily (OD) post thyroidectomy two years ago and dyslipidemia (on rosuvastatin 10 mg OD).

Upon presentation, the patient was afebrile, with a blood pressure of 144/93 mmHg, saturation of 99% breathing room air, and heart rate of 77 bpm. On physical examination, patient had a Glasgow Coma Scale (GCS) score of 15, with no petechiae or rashes, no neurological deficits, normal cardiopulmonary examination, soft abdomen, and no lower limb edema. Point-of-care ultrasound (POCUS) showed no findings of cholecystitis or perforated viscus.

Initial laboratory results (Table 1) revealed thrombocytopenia at 19,000/mm^3^ (reference range of 130,000-400,000/mm^3^), hemoglobin level at 12 mg/dL (12-16 mg/dL), mean corpuscular volume (MCV) of 92 fL (81-95 fL), fibrinogen of 365 (180-350 mg/dL), decreased haptoglobin < 0.1 g/L (0.3-2 g/L), normal PTT and prothrombin time (PT) and international normalized ratio (INR), elevated total bilirubin of 2 mg/dL (0-1.3 mg/dL) with indirect bilirubin of 1.8 mg/dL (0-0.3 mg/dL), lactate dehydrogenase (LDH) of 2,032 IU/L (91-248 IU/L), troponin T hs of 44 ng/L (0-14 ng/L), C-reactive protein (CRP) of 32.3 mg/L (0-6 mg/L), and a creatinine of 1.63 mg/dL (0.44-1 mg/dL). Anisopoikilocytosis with schistocytes (4-5/hpf) were visualized on peripheral smear, and the direct Coombs result was negative.

**Table 1:** Patient's initial laboratory finding The patient's (67, female) laboratory results showing hemolysis, thrombocytopenia, and cardiac myocyte injury.

Laboratory exam	Patient values	Reference range
Platelets	19,000/mm^3^	130,000-400,000/mm^3^
Hemoglobin	12 mg/dL	12-16 mg/dL
Mean corpuscular volume (MCV)	92 fL	81-95 fL
Fibrinogen	365 mg/dL	180-350 mg/dL
Haptoglobin	< 0.1 g/L	0.3-2 g/L
Total bilirubin	2 mg/dL	0-1.3 mg/dL
Indirect bilirubin	1.8 mg/dL	0-0.3 mg/dL
Lactate dehydrogenase (LDH)	2032 IU/L	91-248 IU/L
Troponin T hs	44 ng/L	0-14 ng/L
C-reactive protein (CRP)	32.3 mg/L	0-6 mg/L
Creatinine	1.63 mg/dL	0.44-1 mg/dL

Urine analysis showed numerous amorphous urate crystals, numerous leukocytes, and absent urobilinogen. Cardiac ultrasound and electrocardiogram showed no findings of ischemia. A presumptive diagnosis of TTP was made based on hemolytic anemia and thrombocytopenia, with a PLASMIC (Platelet count; combined hemoLysis variable; absence of Active cancer; absence of Stem-cell or solid-organ transplant; MCV; INR; Creatinine) score of 6 (Table 2) predicting a 72% risk of severe ADAMTS13 deficiency (activity level <15%).

**Table 2:** PLASMIC score PLASMIC (Platelet count; combined hemoLysis variable; absence of Active cancer; absence of Stem-cell or solid-organ transplant; MCV; INR; Creatinine) score of 6 indicating that the patient belongs in a high risk group with a 72% risk of severe ADAMTS13 deficiency. Patients in high risk groups have a significant improvement in the overall survival associated with plasma exchange. MCV: mean corpuscular volume; INR: international normalized ratio

Variable	Points earned if present	Points earned by our patient
Platelet count < 30 x10^9^/L)	+1	+1
Hemolysis	+1	+1
No Active Cancer (treated within the past year)	+1	+1
No history of solid organ or stem cell transplant	+1	+1
MCV (<90 fL)	+1	0
INR (<1.5)	+1	+1
Creatinine (<2 mg/dL)	+1	+1

**Figure 1:**
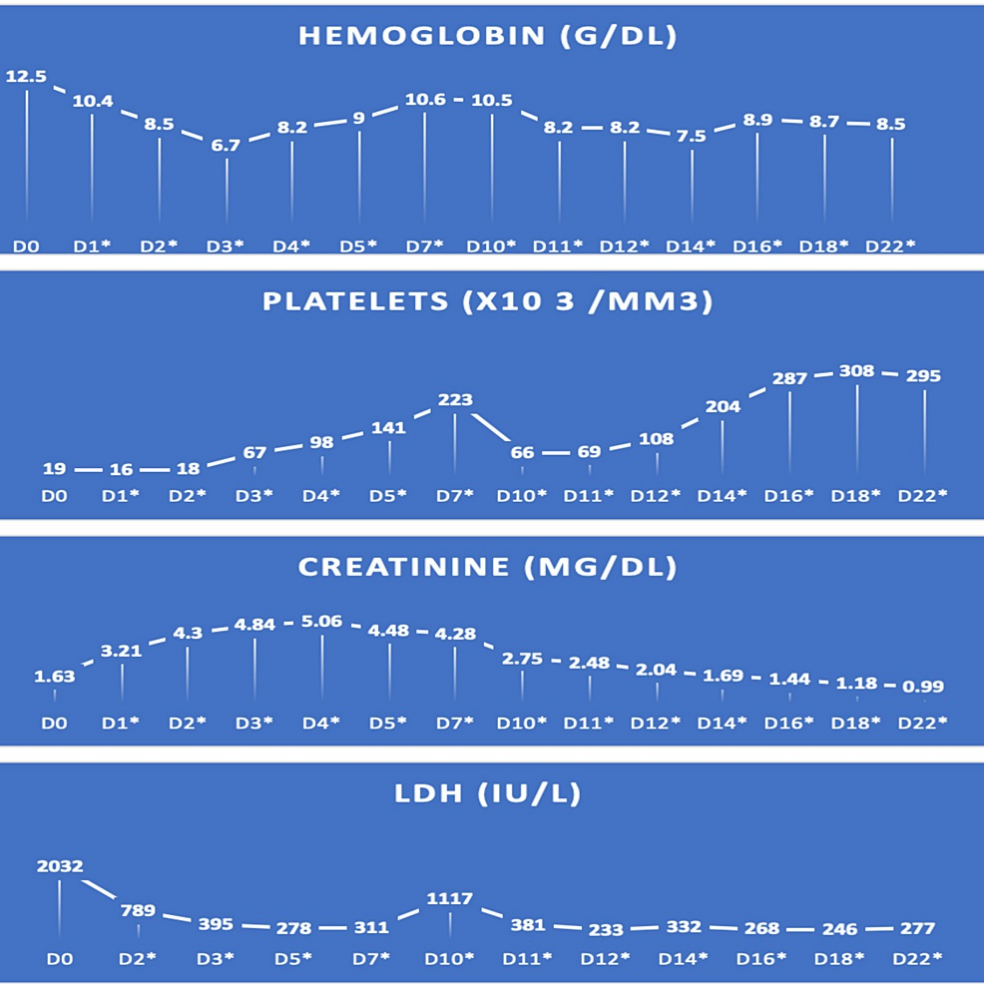
Laboratory results of the patient Progression of hemoglobin, platelets, creatinine, and LDH throughout the hospital course. Days with * refer to the days when therapeutic plasma exchange (TPE) sessions were done.

Subsequently, a central venous subclavian line was inserted, and the patient was started on prednisone 1 mg/kg/day and daily therapeutic plasma exchange (TPE) while being closely monitored in the cardiac care unit. On the following day, platelets dropped to 16,000/mm^3^, hemoglobin dropped to 10.4 mg/dL reaching 6.7 on day four, and creatinine increased to 3.21 mg/dL reaching a peak of 5.06 mg/dL on day five, with oliguria and acidosis responsive to fluids and diuretics. Platelets and LDH reached near-normal levels after five sessions of plasmapheresis (Figure 1).

Due to the patient's clinical and hemodynamic stability and improvement in her biologic findings, TPE was discontinued for three days after the sixth session, after which platelet count decreased to 66,000/mm^3^ and LDH increased to 1117 IU/L. Both parameters improved significantly after re-introducing TPE on the same day.

She received a total of 13 sessions of TPE, after which platelets count, LDH, and creatinine normalized. Additionally, the patient received two doses of rituximab at 375 mg/m^2^ separated by 14 days each.

ADAMTS13 result, drawn before the first session of TPE but reported on day 23 of presentation, showed a very low ADAMTS13 activity of 0.03 IU/mL, and antibodies against ADAMTS13 were not detected, which confirmed the diagnosis of TTP.

After 30 days of hospitalization, the patient was discharged home with close follow-up for three months. Repeat cell count, creatinine, and LDH once weekly and repeat ADAMTS13 levels once monthly showed stabilization of the patient’s condition.­­­­

## Discussion

TMAs are diverse disorders characterized by microvascular thrombi causing a clinical triad of thrombocytopenia, microangiopathic hemolytic anemia, and end-organ ischemia [5]. The thrombotic tendency in TTP results from accumulation of prothrombotic ultra-large multimers of von Willebrand factor (VWF), subsequent to absent or reduced VWF-cleaving protease ADAMTS13 activity [6]. Therefore, end-organ damage caused by TTP often manifests as neurological abnormalities, as well as cardiac or mesenteric ischemia, with neurological complaints dominating the clinical picture, seen in 44% of patients as the first manifestation of TTP [7]. However, severe acute kidney injury, which is a hallmark of HUS, is less commonly described in TTP cases and usually only seen on biopsies [8].

Classically, TTP is recognized by the pentad of fever, thrombocytopenia, microangiopathic hemolytic anemia, neurologic abnormalities, and renal failure. It was recently shown that this symptomatic pentad is neither sensitive nor specific, as the majority of patients diagnosed with TTP do not experience all five clinical features [9], and delay of diagnosis in case of absence of the pentad leads to delays in timely treatment and increase in mortality and morbidity. 

Our patient did not have any neurological symptoms, fever, or purpura. Her symptoms of epigastric pain, anorexia, and nausea can be attributed to the uremia caused by the acute kidney injury. Additionally, further adding to the confusion was that our patient was an older woman, and TTP is usually seen in adults between 20 and 50 years, with one study showing worse outcomes in older patients [10].

In a retrospective study of 46 cases of TMA, Coppo et al. [11] compared two groups of patients on the basis of ADAMTS13 activity (undetectable or detectable) in an attempt to better characterize TTP patients: patients with severe ADAMTS13 deficiency were characterized by mild renal involvement as opposed to patients with detectable ADAMTS13 activity.

In a prospective cohort of 142 patients with clinically diagnosed TMA, Vesely et al. [12] showed a lower frequency of acute kidney injury in patients with severe ADAMTS13 deficiency relatively to other patients. However, in a retrospective study of 92 cases of TTP, Zafrani et al. [13] identified 58.7% of patients with acute kidney injury, including 45.3% with stage 3 acute kidney injury and 25.9% requiring renal replacement. These authors considered that renal involvement was used to differentiate TTP from HUS before ADAMTS13 activity test was available, which led to significantly underreported cases of TTP with severe acute kidney injury [14].

Our patient presented for thrombocytopenia, hemolytic anemia, and uremia with acute kidney injury. She was found to have very low ADAMTS13 activity; therefore, severe acute kidney injury should not rule out TTP or favor HUS in a patient with TMA features.

## Conclusions

TTP is classically defined by the association of microangiopathic hemolytic anemia, thrombocytopenia, fever, and neurological and renal manifestations. However, studies have shown that only 5% of patients present with all pentad combined. That is why a high clinical suspicion index should be maintained. Kidney involvement in TTP is quite rare, and in our case, it was the main manifestation of TTP. Timely management and early initiation of therapeutic plasma exchange saved the patient from progression of the disease to chronic renal failure and high morbidity and mortality.
